# Application of Data Mining to “Big Data” Acquired in Audiology: Principles and Potential

**DOI:** 10.1177/2331216518776817

**Published:** 2018-05-31

**Authors:** Joseph C. Mellor, Michael A. Stone, John Keane

**Affiliations:** 1 School of Computer Science, University of Manchester, UK; 2 Manchester Centre for Audiology and Deafness, University of Manchester, UK; 3 Manchester Academic Health Sciences Centre, University of Manchester, UK; 4 Manchester Institute of Biotechnology, University of Manchester, UK

**Keywords:** audiogram, auditory ecology, big data, candidature, hearing aids

## Abstract

The ubiquity and cheapness of miniature low-power sensors, digital processing, and large amounts of storage contained in small packages has heralded the ability to acquire large amounts of data about systems during their course of operation. The size and complexity of the data sets so generated have colloquially been labeled “big data.” The computer science field of “data mining” has arisen with the purpose of extracting meaning from such data, expressly looking for patterns that not only link historic observations but also predict future behavior. This overview article considers the process, techniques, and interpretation of data mining, with specific focus on its application in audiology. Modern hearing instruments contain data-logging technology to record data separate from the audio stream, such as the acoustic environments in which the device was being used and how the signal processing was consequently operating. Combined with details about the patient, such as the audiogram, the variety of data generated lends itself to a data mining approach. To date, reports of the use and interpretation of these data have been mostly constrained to questions such as looking for changes in patterns of daily use, or the degree and direction of volume control manipulation as the patient’s experience with a hearing aid changes. In this, and an accompanying results paper, the practical applications of some data mining techniques are described as applied to a large data set of examples of real-world device usage, as supplied by a hearing aid manufacturer.

## Introduction

Data mining is the discovery and extraction of patterns and knowledge from large or complex data sets. This covers a wide variety of tasks including grouping or clustering, discovery of dependencies, and detection of anomalous examples within the data. With easier accessibility to larger amounts of data, there has been a greater focus on how to effectively make use of that data and adopt tools and processes that systematically provide new insight, where possible, into the relationships between data. This paradigm shift in data generation and availability has been termed *big data*, which is often characterized by the five Vs (Demchenko, Grosso, de Laat, & Membrey, 2013; Gandomi & Haider, 2015; Ishwarappa & Anuradha, 2015):

1. *Volume*: *This refers to the quantity of the data. The volume may improve the power of methods to find complex patterns within the data*. However, how to process large-scale data can also present a challenge.
2. *Velocity*: *The rate at which data are generated and moved around*. Data with high velocity can present further challenges over data that are static. However, a static history of high-velocity data (“snapshots”) provides a time series where temporal patterns can be learned.
3. *Variety*: *This refers to the different types of data available*. This could range from audiograms, to microphone input levels, to text-based medical records, and beyond.
4. *Veracity*: *How accurate the data are.* For instance, there may be a large measurement noise from a sensor.
5. *Value*: *The cost of obtaining and processing the data compared with the effectiveness of the outcomes obtained*. Data are only as good as the outcomes that arise from it. It is important to apply data mining to uncover patterns that have, as yet, gone unnoticed. However, no amount of data will help if they are of the wrong type or there is no pattern to discover.

Gatehouse, Naylor, and Elberling (2006a, 2006b) showed that benefit from a hearing aid fitting depended on factors measured in other domains, separate from just that of hearing. Data mining may uncover interdomain dependencies when applied to broader ranging data that contain sufficient variety. There are now a large number of fitting options and features. One issue within the field is to evaluate which features lead to benefit for the user, and under what circumstances. As typified by Gatehouse et al. (2006a, 2006b), many laboratory studies, despite their precision and extensive data collection, employ small numbers of participants, leading to low statistical power. Many studies are performed under acute conditions where it is not possible to test the devices in real-world scenarios, despite evidence for longer term acclimatization effects (Gatehouse, 1992). Across multiple studies, the details of implementation vary quite considerably, leading to null or even seemingly contradictory conclusions. In a meta-analysis of self-reported hearing aid use covered by 11 papers over an 11-year span, a wide variety of distributions of hearing aid usage were observed, despite sample sizes varying between 76 and 8,707 participants (Solheim & Hickson, 2017). Many factors varied between the studies, such as age and follow-up time after hearing aid fitting. The data mining approach can be used to search for relationships between these, and other factors that may influence pattern of use, but the technique requires large numbers of data points. Modern hearing prostheses have data-logging facilities that record not just the aid settings and patient details, but how the aid is being used and how it is responding. This sort of information can be collected by manufacturers from their user base. Such data collection comes with both advantages and disadvantages over the more controlled studies. The scale of the logging data means that there is potential to find patterns of use for which a smaller study would lack power. The data are also likely to capture more realistic usage patterns from the user, as well as more realistic patterns of fitting by the practitioner, than would a controlled study. Data mining therefore provides a potentially useful set of tools to uncover important relationships in this type of data.

In this article, we present an overview of a process called Knowledge Discovery in Databases (KDD; Fayyad, Piatetsky-Shapiro, & Smyth, 1996; Han, Kamber, & Pei, 2011) that describes a series of steps to be taken in the general process of converting data into knowledge. A series of sections expand on the salient points of each step. The “Preprocessing” section briefly discusses data cleaning; then, the “Data Mining Techniques: Examples” section introduces common data mining techniques and shows an example method for each. These techniques include clustering, classification, and regression; within each, we give an example of how the method may be of relevance to the field of audiology. As Sullivan (2011), and many others, point out, data mining is not a panacea; there is potential for many spurious patterns to be returned by the tools, and so care must be taken in the interpretation of results in order to provide their proper context. The “Interpretation/Evaluation” section therefore discusses the generation of value from the data mining. Any statistically significant relationships that have been revealed between data members need to be sifted by an expert in the field in order to sort the spurious from those that generate insight.

## The KDD Process

The KDD process used to acquire knowledge from data involves the following steps:

1. *Selection*: Selecting a data set with a subset of variables or data samples. Selection is guided by prior domain knowledge and end-user goals.
2. *Preprocessing*: Cleaning the data set; this includes removing outliers or noise and handling missing data.
3. *Transformation*: The data are further transformed into a form more useful for the data mining task; this can include reducing the number of feature variables to the most relevant, or projecting the features to a more useful space, such as a logarithmic rather than a linear scale.
4. *Data mining*: Applying the appropriate task and method to the data; tasks include Classification, Regression, Clustering, and Subgroup Discovery.
5. *Interpretation/Evaluation*: Task-dependent evaluation of the patterns learned via data mining; domain knowledge is used to assess whether these patterns make sense with respect to the domain to avoid spurious results.

**Figure 1.**
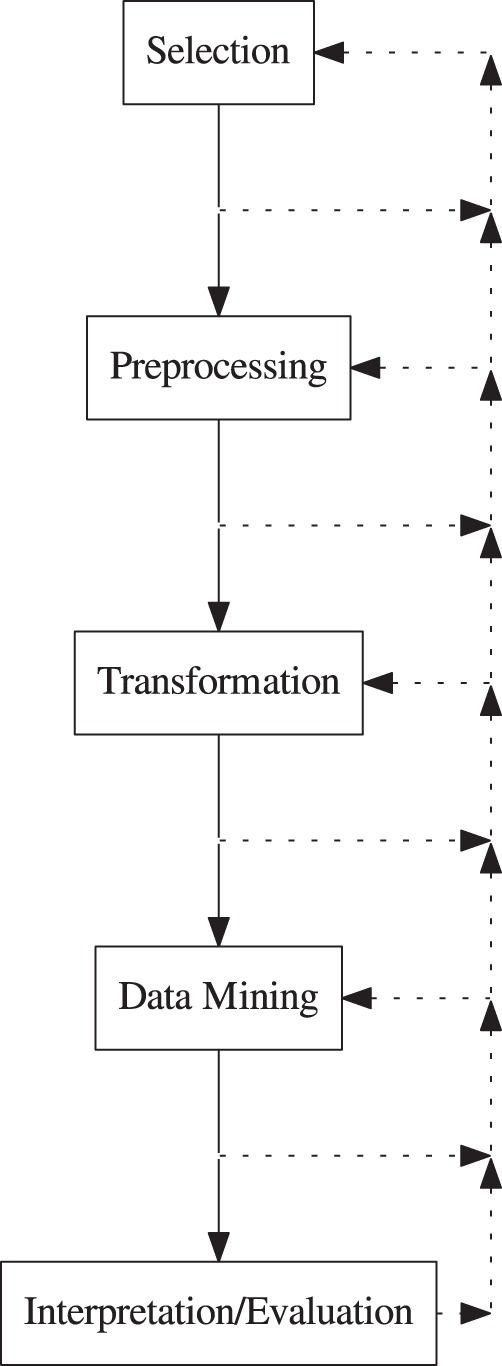
Stages in the KDD process. See text for details. KDD = Knowledge Discovery in Databases.

As Figure 1 illustrates, the process is not necessarily unidirectional between the separate stages, and ultimately the entire process may involve elements of iteration in order to build confidence in the results from the discovery process.

## Preprocessing

The data collected can be noisy or anomalous in a multitude of ways. For example, values in some part of the data set may be missing. Often, records with missing values are simply dropped for a specific analysis. However, missing values can sometimes be imputed. Young, Weckman, and Holland (2011) provide a survey of such techniques. Records that are either obviously wrong or unreliable may need to be filtered out before a full analysis can begin. These are two theoretical examples of the iteration required within the preprocessing stage of KDD.

Practical examples of missing, wrong, or unreliable data come from consideration of measurements of an audiogram. Even with a complete audiogram, that is, no missing values, one may doubt the veracity (the fourth “V” described earlier) of some or all of the recorded values. This could be for several reasons, such as a nonorganic hearing loss, operator error, or the audiogram values being deliberately adjusted from their true value for purposes best known to the clinician. A second source of doubt could arise in the velocity (the second “V” described earlier) recorded in a data set. Successive visits of a device to a clinic may prompt a repeat measure of the audiogram, thereby creating a time series of audiograms. There is an implicit assumption that this time series has been generated from or by the same wearer. Large changes recorded between successive visits may have a valid explanation, such as a fluctuating hearing loss. However, the pattern of changes may also indicate that the device has been loaned temporarily at different times to multiple wearers. With this interpretation, a time series does not represent the experience of a unique wearer of the aid, could lead to error-filled analyses, and so may become a candidate for either separate handling or complete removal.

## Transformation

Although transformation can take many forms, such as arithmetic manipulation of data values, a general aim in its use could be to ensure that any one data dimension/feature does not dominate, thereby introducing a bias to the results. Arithmetic transformations are regularly used in the field of audiology: The decibel represents a logarithmic scaling of sound pressure level, while standard audiogram test frequencies are at spacings of one octave, a ratio transformation. The reason for doing so is that the spacing of the scale units are chosen to correspond approximately to a similar degree of perceptual distance, across a wide range of the scale (1,000,000 to 1 in pressure, and 1,000 to 1 in frequency). In data mining, a common transformation is to scale the data features so that, within each data type, statistically, they have a mean of zero and variance of unity, using what is known as the *z*-score transform.

## Data Mining Techniques: Examples

We now discuss the following data mining methods: Clustering, Subgroup discovery, Classification, and Regression.

### Clustering

Clustering is a common task in exploratory data mining. It is an unsupervised learning task to identify meaningful groupings of the data into classes that are not known beforehand (“a priori” or “prior”) but instead are learned from the data. With clustering, points in the data set are grouped such that points within a given group are more similar to each other, in some sense, than points outside of the group. Such exploratory data mining is useful in our context as it may help uncover common profiles of hearing or lifestyle. Audiograms, for instance, can be clustered so that audiograms of similar shape are assigned into the same group (Lee, Hwang, Hou, & Liu, 2010). Summary statistics of the learned clusters can often give a more informative, high-level, view about the composition of the users, and possibly even etiologies. In addition, such clustering may promote selection of a particular device tailored to better fit common profiles, for example, reverse slope when compared with presbyacusis audiograms. With a new-to-market device, as the associated database grew, such a selection could be verified by comparing outcome measures across devices. The concept of a “good” (as in sensible and robust) grouping can vary quite considerably, and so there are numerous clustering algorithms in the data mining literature. A simple, yet often effective, workhorse for clustering is K-means (Wu & Kumar, 2009, pp. 21–33). K-means is a method to find a number, K, of clusters such that the sum of the variance of all of the clusters is minimized. This is done with respect to some metric space, which is normally Euclidean. An equation defining the objective function of K-means clustering is given in the Supplementary Material.

### Subgroup Discovery

The aim of this subgroup discovery is to search for interesting subgroups within the data set for some predetermined notion of “interestingness.” A practical translation of this is as follows: “Are there patterns of behavior (i.e., interrelationships in the data) that are far more, or far less, common than would occur by chance.” By adjusting for the size of data subsets, we ensure that no parts of the data set have an overrepresentation in any patterns found. Caution needs to be exhibited in overinterpreting significant links: Due to the large search area for possible links, there may be spurious subgroupings present so any results need expert interpretation, hence Stage 5 of the KDD process listed earlier.

Gatehouse et al. (2006b) identified the speed of dynamic range compression producing different patterns of benefit depending on the lifestyle of the aid wearer. “Lifestyle” was defined as the range of auditory environments in which the wearer operated, which was characterized by more than just the mean sound level. For example, an extra characterization was by the range of sound levels encountered, not just on a moment-to-moment basis, but across days of the week. This linkage between benefit (speech-in-noise scores) and multidimensional measures of the auditory environment indicated that candidature was multifactorial. Subgroup discovery in data mining could permit more subtle relationships to be elucidated. For example, the number of programs activated could be determined by linking in other factors from the patient’s medical records, such as dexterity problems. Alternatively, when attending a fine-tuning session, the data records of sound levels encountered, as well as the proportion of time spent in each sound category (e.g., quiet, noise, music), may show a different lifestyle from that previously recorded. Because the wearer has moved between subgroups, the previous settings could then be no longer optimal, and the fitting software could recommend new settings. In addition, the concept of “benefit” could be widened from the consideration of conventional measures, such as intelligibility or questionnaires, to include indicators of lifestyle changes, such as those inferred by a more active lifestyle.

The grouping of dimensions/features available from hearing aid data logging can be referred to as a modality (refer to the Glossary for a more rigid definition; Lahat, Adali, & Jutten, 2015). It is common in data mining to search for patterns (relationships) between modalities *X* and *Y* such that Y=f(X) for some unspecified function *f* that must be learned from the data. For example, the way that an increasing degree of hearing loss may restrict lifestyle, as reflected by, say, a measure of how often “speech-in-noise” is detected by the sound-environment classifier. The pattern is usually global such that all examples of *X* map to some value of *Y*. However, there are many useful patterns that do not exhibit this global form because they comprise a minority in the data set. Consequently, an interesting pattern may only be exhibited in a subgroup of the data. Large data sets enable these subgroups to become of sufficient size that their patterns become significant and stand out from the “noise” that is inherent in many data collection exercises. Without data mining, these patterns would be ignored.

These patterns are where the data “clusters” due to the similarities between the group members. There may be many, or few, clusters detected, depending on the selection criteria, such as requiring that a cluster stands sufficiently far above the noise to merit attention. When there are many clusters selected, the differences between clusters may appear small to the human observer. In our companion paper (Mellor, Stone, & Keane, 2018), we chose an arbitrary number of clusters, usually five as a “proof-of-concept” such that the patterns in the outputs of the analyses are more obvious to the reader. There may be many more, or even fewer, in real-world data sets.

Modalities are often highly structured, such as with the clinical audiogram. This is usually recorded at octave frequencies between 250 and 8000 Hz, as well as at 3000 and 6000 Hz, a total of eight values, to form a multidimensional object (commonly with high correlation of values at adjacent frequencies). A second example of a modality would be that, although the sound levels of the environments in which the aid was used form a continuous range, the logging of aid operation may be quantized into a fixed number of levels by grouping a range of levels, such as in steps of 1, 2, or 5 dB. In comparison, unstructured modalities can be generated from open-ended data such as patient reports, where the dimensions of data are more flexible.

For data mining of relationships between structured modalities such as the audiogram, one approach was proposed by Umek and Zupan (2011). Let our data set be called *D*. The “∈” symbol is shorthand for “is a member of.” The “∪” symbol is shorthand for “the sum of.” Let *C_x_* be a set of clusters in the modality *X*, and let *C_y_* be a set of clusters in the modality *y* such that $\underset{c_{x}\in C_{x}}{⋃}c_{x}=D$ and $\underset{c_{y}\in C_{y}}{⋃}c_{y}=D$. That is, the clusters found in *C_x_* and *C_y_* contain the entire data set. Let $c_{x}\in C_{x}$ be a cluster in the modality *X*, and let $c_{y}\in C_{y}$ be a cluster in the modality *Y*. All examples that belong to both *c_x_* and *c_y_* form a subgroup. In audiology, this could be a subgroup of hearing devices, or human wearers, because devices are (usually) assumed to collect data when attached to a wearer (hence the need for preprocessing to identify and remove most of the cases of “aid left switched on and sitting in a box”). We assume a subgroup is interesting if the size of a subgroup is larger than would be expected if the clustering *c_x_* was independent of *c_y_*. This can be checked using a statistical test where we accept or reject the hypothesis with a given confidence level. When considering clusters *c_x_* and *c_y_*, there are four counts to consider: (a) the number of examples in both *c_x_* and *c_y_*, (b) the number of examples in *c_x_* but not in *c_y_*, (c) the number of examples in *c_y_* but not *c_x_*, and (d) the number of examples in neither *c_x_* nor *c_y_*. This leads to a 2 × 2 contingency table, where an appropriate statistical test is the $\chi^{2}$ test. In this method, because we are performing multiple hypothesis tests, we need to correct for the possibility of false positives. A simple approach to do this is via the Bonferroni correction (Haynes, 2013), where the confidence level required to denote significance is scaled by the number of tests performed. We can further refine the search for interesting patterns by ensuring that the estimate of the “effect size,” and the size of the subgroup, both exceed a certain threshold so as to eliminate effects of low relevance or remote chance of occurrence.

We take the effect size to be the ratio of the joint probability of clusters *c_x_* and *c_y_* and the product of the marginal probabilities of *c_x_* and *c_y_*. The marginal probability can be expressed in terms of the joint probability as follows:

(1) $$P(c_{x})=\sum_{c_{y}\in C_{y}}P(c_{x},c_{y})$$

It is the probability of one variable having a given value without knowledge of the value of any other variable.

Because these probabilities are not known a priori, they are estimated from the data. The estimated effect size *E* is given by *N* times the ratio of the number of examples in *both* cluster *c_x_* and *c_y_* to the product of the number of examples in each cluster separately.

**Figure 2.**
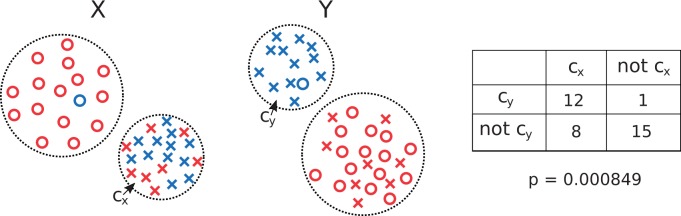
Example of subgroup discovery. In this example, the data are clustered into two clusters in both modality *X* and *Y*. The two clusters of modality *X* are shown on the left of the figure with one cluster containing crosses and the other circles. The two clusters of modality *Y* are shown in the center of the figure with one cluster containing blue points and the other red points. We consider the subgroup made from *C_x_* and *C_y_* (marked on figure). The right of the figure shows the contingency table produced and the *p* value from a $\chi^{2}$ test associated with the table. From this, we would conclude that the subgroup formed by *C_x_* and *C_y_* is interesting, and there is a dependence between the two modalities.

Outline pseudocode for the procedure is given in the Supplementary Material in Algorithm 1. A simple example is shown in Figure 2 where the “interestingness” is that cluster *c_x_* contains only crosses, and cluster *c_y_* contains only blue data points.

### Classification

Classification is a supervised learning task. In contrast to clustering, meaningful groupings of the data are known a priori and are provided as labels. The task is to be able to accurately predict the labels, given the data. Given a data set $X=\{x_{1},\ldots ,x_{N}{\}}^{T}$ of *N* examples and associated categorical labels $y=\{y_{1},\ldots ,y_{N}{\}}^{T}$, classification is the task of finding a mapping from *X* to **y**. The mapping can be used to predict a new label $y_{N+1}$, given a new example $x_{N+1}$. This mapping is chosen to minimize the prediction error. As an example, from a hearing aid manufacturer, there may be a fixed number of styles of device available (completely-in-the-canal, in-the-ear, behind-the-ear, etc.), and the most basic data consist of the audiograms for users of these devices. For existing users, we know which style of device the user has (ideally further qualified by some measure of benefit), and so we can analyze to see whether particular audiogram patterns are associated with each style of device. A simplistic task would then be to build a classification model to predict which style is most likely to be suitable for a new user on the basis of their audiogram alone. In this example, for didactic purposes only, we have ignored some of the more real-world qualifiers that may further influence device choice such as the dexterity of the user or cost.

**Figure 3.**
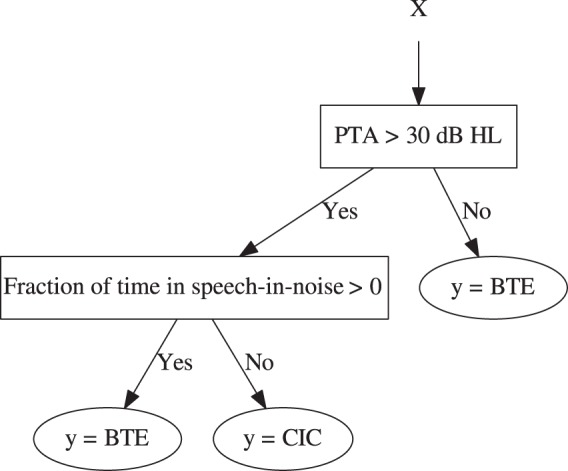
Example decision tree. The input data point, *x*, enters at the top of the tree. The example enters a decision node and is directed down the tree to the relevant branch (or “child”) where it can either enter another decision node or reach a leaf node. This process repeats until a leaf node is reached. The leaf node states the prediction *y* for the input *x*. The actual decision nodes shown are illustrative and not intended to represent a practical classifier. The transparency and understandability of the associated reasoning of the “decision” made (the output classification) should be clear; furthermore, a set of rules can be generated from a decision tree that uses domain- and data set-specific vocabulary. PTA = pure tone audiometry; BTE = behind-the-ear; CIC = completely-in-the-canal.

One successful classification model is the Random Forest (Caruana, Karampatziakis, & Yessenalina, 2008; Robnik-Šikonja, 2004; Wyner, Olson, Bleich, & Mease, 2017). Breiman (2001b), the inventor of Random Forests, called them “A+ predictors.” A Random Forest employs multiple decision trees (Breiman, 2001a). Each decision tree functions as a classifier, or regressor, based on decision rules expressed within a tree-like structure; the branch structure develops as a product of the dimensions available from obeying each of a string of rules. An example decision tree is shown in Figure 3. The final decision of the branching is represented by a leaf. Therefore, the decision from a Random Forest analysis is the aggregated decision of all the individual trees within the forest and so represents a “best of multiple estimates” rather than just a single estimate from the available data.

The structure of each tree in the forest is defined by the results generated from a set of training data via “bagging.” Bagging (or **b**ootstrap **agg**regat**ing**) is a procedure of sampling with replacement from the training set. In addition, the candidate dimensions for splitting at each node are a random subset of the total set of dimensions. The generation of each tree in the forest by the use of bagging and random subsets of candidate dimensions decreases the statistical dependence between trees, thereby improving the estimate of the final, aggregated, decision from the forest.

The decision that splits the data at each node is chosen to optimize some measure; a common measure used is the “Gini impurity.” Given a random relabeling of the data, where the labels are sampled from the distribution given by the proportion of labels at the node, the Gini impurity is a measure of how often the random label would mismatch the true label. The smaller the value of the Gini impurity, the better the decision separates the data with respect to the labels. It is zero when there is only one category in the node. An equation defining the Gini impurity is given in the Supplementary Material. The training data are partitioned or clustered by the learned decision tree, with each leaf representing a partition or cluster. Random forests are robust to the scaling of data, and so transformations of the data can be less important than in other methods. A more in-depth discussion of decision trees and Random forests can be found in Flach (2012). An implementation of Random forests is provided by the scikit-learn python library (Pedregosa et al., 2011).

### Regression

Regression is similar to classification (see Classification subsection described earlier) except that the labels $y=\{y_{1},\ldots ,y_{N}{\}}^{T}$ are not categorical but instead are continuous real valued. For example, whereas classification is appropriate for predicting the type of device (behind-the-ear, completely-in-the-canal, etc.), regression is an appropriate method for, for example, predicting the absolute threshold for a given audiogram frequency.

### Gaussian Process Regression

Gaussian processes are a popular model that can be used for both classification and regression tasks. They are a probabilistic model, and so one of their great strengths is in quantifying uncertainty. That is, not only will the model provide a prediction, but it can also provide a measure of confidence in the prediction. Gardner et al. (2015) exploited this aspect of Gaussian processes to propose a new audiogram estimation technique that can significantly reduce the time required to measure an audiogram. One practical application in audiology is that the uncertainty measure can be useful in detecting outliers within a data set: The outlier represents an (highly) unexpected setting or operation that may warrant further investigation as to the cause.

**Figure 4.**
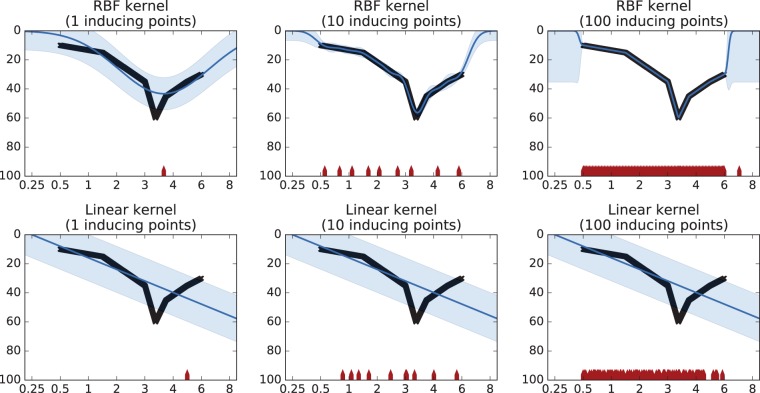
An example of Gaussian process regression to model a data set similar to an audiogram. The abscissa in each plot can be taken as frequency in kHz, while the ordinate represents threshold in dB(HL). The blue lines show the mean prediction of the Gaussian process, and the shaded blue areas show the associated confidence regions. The top plots show the use of a squared-exponential (RBF) kernel, and the bottom plots show a linear kernel. The leftmost plots show the use of a single inducing point, depicted by a red marker on the *x*-axis. The middle plots show the use of 10 inducing points, and the rightmost plots show the use of 100 inducing points. The solid black line shows the function to be approximated. Use of either an inappropriate number or spacing of inducing points, or insufficiently flexible kernel, leads to poor fitting (blue line lying outside of the black line). RBF = radial basis function.

Here, we provide a brief overview of Gaussian processes with respect to the regression task. A Gaussian process is a collection of random variables where the joint distribution of any finite subset of the collection is a multivariate Gaussian distribution (Rasmussen & Williams, 2006). A Gaussian process is defined by a mean function, m(x), and covariance function, k(x,x') and can be thought of as a prior distribution over functions. For simplicity, it is often assumed that m(x)=0. Given this prior knowledge, and observations X with labels **y**, the rules of probability can be applied to provide an a posteriori prediction of a label $y_{*}$ for a new observation $x_{*}$ (a “posterior”). Assuming Gaussian noise on the observations and a Gaussian process prior on the function to be learned, the posterior is also given by a Gaussian process. That is, for a new input, the Gaussian process provides a distribution of potential values. This distribution provides an estimate of the uncertainty for the predictions made. The values of the diagonal of the covariance (the variance) of the posterior give an indicator of how uncertain is the prediction for that input. The lower the variance, the more confident is the prediction. Equations for defining the posterior are given in the Supplementary Material, while Rasmussen and Williams (2006) give a thorough treatise of Gaussian processes. The form of the covariance function determines the type of functions that are considered. For instance, the linear covariance function can be used to model linear functions, and the squared-exponential (“radial basis function”) covariance function can be used to model smoothly varying functions. These covariance functions can even be combined through addition or multiplication. For instance, a Gaussian process with a covariance function that is the sum of a linear covariance function and a squared-exponential covariance function might model a smoothly varying function to be approximated that has a general linearly increasing trend. The computational cost of obtaining the posterior distribution is of the order of *N*^3^, where *N* is the number of examples in the data set. For large data sets, this is prohibitively expensive. To reduce the computational cost of the model, sparse Gaussian processes (Hensman, de G Matthews, & Ghahramani, 2015) can be used which have an order of $({\mathrm{NM}}^{2})$ complexity where *M* is the number of “inducing points.” Inducing points can be thought of as an alternative, reduced size, data set, which are chosen such that the posterior obtained using these alternatives closely approximates the original data set. As long as the number of inducing points, *M*, is small and appropriately chosen, then the method can be applied to very large data sets. The GPy python library provides a comprehensive set of Gaussian process implementations (The GPy authors, 2012–2015). An illustration of the effects of the number and spacing of inducing points, as well as the choice of kernel, is given in Figure 4. This could be for a data set comprising “all those with a claim of noise-induced hearing loss.” The use of a radial basis function as the regression kernel (top row of panels), and a modest number of inducing points (middle panel), compared with a linear regression (bottom row of panels), gives rise to an accurate fit to the data. Within a data set of such audiograms, if a particular audiogram lay well outside of the confidence regions (drawn particularly tightly in this example), then, it could be flagged as “anomalous,” and the cause for such be investigated (e.g., nonorganic loss, uncalibrated equipment, or transcription error). As such, this is a fairly trivial example, but extension of this technique could identify an individual’s pattern of usage differing from that expected from the other members of the user population with a similar set of data, such as degree of hearing loss and age. Again, further investigation as to a possible cause may then be warranted.

## Interpretation/Evaluation

Data mining may offer great promise at finding novel and complex relationships within data sets, but because of the size of the data sets, and the number of comparisons made during mining, many of these may be spurious. Beside statistical confidence, expert interpretation and validation will always be required in order to provide context and to extract potential value from the findings. Unexpected findings, if they can lead to the generation of rational hypotheses, may prompt new areas of targeted research.

Elements of the data set that were either discarded or partly resynthesized in order to overcome missing values may introduce a bias into analyses. Experimental rigor demands the understanding and possible quantification of such bias.

For a task such as classification or regression, the objective is the predictive power of the learned model on new instances of data, which prompts several questions: (a) where does a newly acquired data set fit into the patterns from historic data sets and, if it does not, (b) does the model need updating, and finally, (c) how does that affect our decisions on patient management? A model that does not generalize to being able to obtain sensible predictions from new data, but models only the training data, is called “overfitted” and is comparatively useless.

The performance of a model should therefore be evaluated on data that are separate to the data used to train or update the model. The estimated performance of the model based on the training data will be overconfident because the model can be adapted to fit the seen data specifically and hence may overfit the data. This is analogous to providing a student with the answers ahead of an exam so they can learn them by rote and expect their exam results to provide an unbiased indicator of the student’s knowledge on the general subject.

**Figure 5.**
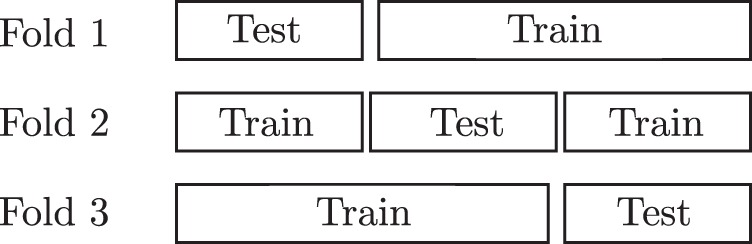
A visualization of splitting data in a threefold cross-validation. Each row shows a different split of the data, where a single fold is used as test data, and the contents of the remaining folds are used as training data for the classification or regression model.

To make efficient use of the data available, while obtaining a less biased estimate of performance, we employ a procedure called “N-fold cross-validation.” A number of folds, *N*, are selected (a common choice of *N* is 10) in order to partition the data into *N* separate subsamples by use of “folds,” divisions of the data set. For each of *N* models to be trained, one subsample, delimited by the fold boundaries, is retained for testing of the model, while the remaining *N*−1 subsamples are used to train the same model. This will produce *N* unbiased estimates of performance. If estimates of performance derived from the training data are high while the testing estimates are low, then the model has overfit the data and has not generalized well. The splitting of data for a threefold cross-validation is visualized in Figure 5.

## Conclusion

We have presented an overview of the field of data mining where we have

1. used the “five Vs” as an outline framework for data properties that are necessary in order to support meaningful analyses;
2. used the (iterative) structure of the KDD process as a template for supporting the acquisition of knowledge from the analyses; and
3. illustrated methods for the tasks of classification, regression, clustering, and subgroup discovery within big data sets.

To demonstrate the potential value from data mining to the field of audiology, we follow up this overview with an application of the described techniques to a large hearing aid manufacturer’s data set (Mellor et al., 2018).

## Glossary

Classification is the task of predicting the label or category of a new observation (from a set of labels or categories), given a training set of data containing observations (or instances) whose labels are already known.

Clustering is the task of grouping observations (or instances) into groups known as clusters, given a training set of data containing observations. The goal is that instances in the same cluster should be more similar to each other than to instances in other clusters. Unlike with classification, no labels are provided beforehand.

Dimension is a synonym for an attribute or feature. An example entry, or instance, in the data set will be described by a set of dimensions. Examples of dimensions are height, gender, and age, or a measure of absolute threshold at a single frequency.

Domain is a high-level modality, where the concept is broader in nature. For example, a person’s lifestyle may be described in a given domain, and their hearing status may be described in another. Each domain can be measured by multiple dimensions/features that may be grouped into multiple modalities.

Modality is a set of related dimensions/features that describe a single object or concept. For example, a clinical audiogram is typically specified by thresholds at eight different frequencies. When the dimensions together describe a single concept, such as an audiogram, we term this a modality.

Regression is the task of predicting the continuous response to an input variable, given a set of training data containing observations whose continuous response is already known. This prediction of a continuous response is as opposed to classification where solely a discrete label or category is predicted.

Subgroup discovery is the task of finding a subset of instances in a data set for which some relationship or dependency holds. This is as opposed to classification, regression, and clustering that provide some prediction or description of the *whole* data set.

## Supplemental Material

Supplemental material for Application of Data Mining to “Big Data” Acquired in Audiology: Principles and Potential Click here for additional data file. Supplemental material for Application of Data Mining to “Big Data” Acquired in Audiology: Principles and Potential by Joseph C. Mellor, Michael A. Stone and John Keane in Trends in Hearing
