# Supplementary material for: Application of Data Mining to “Big Data” Acquired in Audiology: Principles and Potential
Source: Trends Hear. 2018 May 31;22:2331216518776817. doi: 10.1177/2331216518776817 (PMC6022814; doi:10.1177/2331216518776817)
Supplement: Supplemental material for Application of Data Mining to “Big Data” Acquired in Audiology: Principles and Potential [file Supplemental_material.pdf]

Supplemental material

K-means clustering

K-means clustering can be stated as finding a set of K cluster centers $\{\mu_i\}_{i=1}^{K}$ that for a dataset of N examples $\mathbf{x}_j$ satisfies,

$$\arg\min_{\{\mu_i\}_{i=1}^{K}} \sum_{j=1}^{N} \min_{i\in\{1,\ldots,K\}} \|\mu_i - \mathbf{x}_j\|^2.$$

Subgroup discovery

Algorithm 1 Subgroup discovery procedure

```
N - number of examples.
α ∈ (0,1) - size as percentage of total, for subgroup to have significant size.
β > 1 - size of effect for interesting subgroup.
N_x - number of clusters for modality X.
N_y - number of clusters for modality Y.
Perform K-Means on modality X with clusters C_x.
Perform K-Means on modality Y with clusters C_y.
for c_x ∈ C_x do
  for c_y ∈ C_y do
    if reject hypothesis c_x is independent to c_y then
      Classify subgroup of c_x ∧ c_y as interesting.
      if Number of examples in cluster c_x and c_y > αN then
        Classify subgroup as significant in size.
      end if
      if N|c_x ∧ c_y|/|c_x||c_y| > β then
        Classify subgroup effect as significant.
      end if
    end if
  end for
end for
```

Gini impurity

Given data containing N examples each labeled with 1 of J categorical labels, where N_i of the examples are labeled with category i, the Gini impurity is defined as,

$$I_G = \sum_{i=1}^{J} \frac{N_i}{N}\left(1 - \frac{N_i}{N}\right). \tag{2}$$

Gaussian process regression

A Gaussian process is a collection of random variables where the joint distribution of any finite subset of the collection is a multivariate Gaussian distribution (Rasmussen and Williams 2006). A Gaussian process is defined by a mean function, $m(\mathbf{x})$, and covariance function, $k(\mathbf{x}, \mathbf{x}\prime)$ and can be thought of as a prior distribution over functions. For simplicity it is often assumed that $m(\mathbf{x}) = 0$. Given this prior knowledge, and observations X with labels $\mathbf{y}$, the rules of probability can be applied to provide an *a posteriori* prediction of a label y_* for a new observation $\mathbf{x}_*$ (a "posterior"). Assuming Gaussian noise on the observations and a Gaussian process prior on the function to be learned, the posterior is also given by a Gaussian process. Assuming Gaussian noise, the posterior is given by,

$$P\left(y_* | \mathbf{x}_*, X, \mathbf{y}\right) = \mathcal{N}\left(\mu_*, \Sigma_*\right), \tag{3}$$

where $\mathcal{N}()$ is a multivariate Gaussian distribution with mean μ_*, and covariance Σ_*, where,

$$\mu_* = K\left(\mathbf{x}_*, X\right)\left[K\left(X, X\right) + \sigma^2 I\right]^{-1}\mathbf{y}, \tag{4}$$

$$\Sigma_* = K\left(\mathbf{x}_*, \mathbf{x}_*\right) \tag{5}$$

$$- K\left(\mathbf{x}_*, X\right)\left[K\left(X, X\right) + \sigma^2 I\right]^{-1} K\left(X, \mathbf{x}_*\right). \tag{6}$$

Here $K(X, X\prime)$ is the covariance matrix, where the entry $K_{i,j}$ corresponds to the covariance function applied to the i example in X and the j example in $X\prime$, and σ^2 is the measurement noise in the observations. This noise is assumed to be constant. The values of the diagonal of the covariance (the variance) of the posterior gives an indicator of how uncertain the prediction for that input is. The lower the variance the more confident the prediction.
